# Immunogenicity of novel vB_EcoS_NBD2 bacteriophage-originated nanotubes as a carrier for peptide-based vaccines

**DOI:** 10.1016/j.virusres.2024.199370

**Published:** 2024-04-24

**Authors:** Aliona Avižinienė, Indrė Dalgėdienė, Julija Armalytė, Rasa Petraitytė-Burneikienė

**Affiliations:** a Department of Eukaryote Gene Engineering, Institute of Biotechnology, Life Sciences Center, Vilnius University, Saulėtekio av. 7, Vilnius, Lithuania; b Department of Immunology, Institute of Biotechnology, Life Sciences Center, Vilnius University, Saulėtekio av. 7, Vilnius, Lithuania; c Institute of Biosciences, Life Sciences Center, Vilnius University, Saulėtekio av. 7, Vilnius, Lithuania

**Keywords:** Virus-based nanoparticles, Self-assembly, Polytubes, Epitope display, Immunogenicity, *Saccharomyces cerevisiae*

## Abstract

Non-infectious virus-like nanoparticles mimic native virus structures and can be modified by inserting foreign protein fragments, making them immunogenic tools for antigen presentation. This study investigated, for the first time, the immunogenicity of long and flexible polytubes formed by yeast-expressed tail tube protein gp39 of bacteriophage vB_EcoS_NBD2 and evaluated their ability to elicit an immune response against the inserted protein fragments. Protein gp39-based polytubes induced humoral immune response in mice, even without the use of adjuvant. Bioinformatics analysis guided the selection of protein fragments from *Acinetobacter baumannii* for insertion into the C-terminus of gp39. Chimeric polytubes, displaying 28-amino acid long OmpA protein fragment, induced IgG response against OmpA protein fragment in immunized mice. These polytubes demonstrated their effectiveness both as antigen carrier and an adjuvant, when the OmpA fragments were either displayed on chimeric polytubes or used alongside with the unmodified polytubes. Our findings expand the potential applications of long and flexible polytubes, contributing to the development of novel antigen carriers with improved immunogenicity and antigen presentation capabilities.

## Highlights

• Bacteriophage-based polytubes are immunogenic in mice even without adjuvants.
• The polytubes can display OmpA protein fragments from A*cinetobacter baumannii*.
• Chimeric polytubes elicit humoral immune response against Omp28 peptide.
• Unmodified polytubes induce immunogenicity of unconjugated Omp28 peptide.

## 1 Introduction

The self-assembly of viral structural proteins into virus-like particles (VLPs) or virus-based nanoparticles (VNPs) has gained increasing attention in recent years. These non-infectious and replication-deficient multi-subunit nanoparticles mimic the structure of native viruses (Kirnbauer et al., 1992; Mohsen et al., 2020) and can be modified by inserting protein fragments into particle-forming proteins, resulting in versatile nanotools (Carignan et al., 2015; Kalnciema et al., 2011; Zamora-Ceballos et al., 2022). Well-organized surface proteins of viral nanostructures stimulate both humoral and cellular immune responses, leading to an increased immune reaction against protein fragments displayed on nanostructures (Mallajosyula et al., 2014; Ward et al., 2021). For this reason, viral nanostructures have proven to be an effective approach for enhancing protein or peptide immunogenicity in the development of vaccines against viruses, bacteria, cancer, and chronic diseases (Carignan et al., 2015; Govasli et al., 2019; Hawkes, 2015). The success of the use of viral nanostructures has been demonstrated in commercialized vaccines against human papilloma virus (Jochmus et al., 1999), malaria (Hawkes, 2015), human hepatitis B (Wiedermann et al., 1987) and hepatitis E viruses (Wu et al., 2012), and more clinical trials are underway for broader applications of viral nanostructures (Huang et al., 2017; Tariq et al., 2022).

However, the use of genetic fusion to insert foreign protein fragments can significantly affect the chemical and physical properties of viral nanostructures (Karpenko et al., 2000). The insertion of a foreign protein fragment can alter the surface charge, solubility and size of the viral particle, leading to the changes in particle stability, conformation or even in reduction of immunogenicity (Chen et al., 2021; Fan et al., 2015; Frietze et al., 2016; Lawatscheck et al., 2007; Uhde-Holzem et al., 2007). Consequently, there is no universally applicable approach for modifying viral nanostructures without disrupting protein folding, self-assembly or causing protein aggregation. Therefore, the design of protein fragments for the insertion into particular regions within viral particles requires careful consideration of their chemical and physical properties to ensure successful self-assembly of nanostructures (Frietze et al., 2016; Uhde-Holzem et al., 2007). In contrast to icosahedral VLPs, tubular nanoparticles contain larger numbers of scaffold protein copies exhibiting helical symmetry, allowing more efficient display of foreign peptides (Schlick et al., 2005; Wang et al., 2002).

*Acinetobacter baumannii* is a multidrug-resistant pathogen that caused significant nosocomial infections for decades, and with the pandemic caused by SARS-CoV-2, there was an increased risk of ventilator-associated pneumonia caused by this pathogen (Chen et al., 2020; Lai et al., 2020; Rawson et al., 2020). Despite the prioritization by the World Health Organization for developing new medical countermeasures against *A. baumannii*, there is still no licensed vaccine available (Lau and Tan, 2023; Willyard, 2017; World Health Organization, 2017). The biological functions of Blp1 and OmpA proteins are pivotal in *A. baumannii*'s virulence and resistance mechanisms, particularly in biofilm formation, which confers resistance to detergents and antibiotics. The outer membrane protein OmpA, abundantly present in bacterial cell membranes, exhibits high sequence conservation among different *A. baumannii* strains (Badmasti et al., 2015; Luo et al., 2012). Although certain bacterial proteins are concealed beneath a polysaccharide layer, membrane proteins like OmpA remain exposed, facilitating their interaction with the peptidoglycan layer and enabling the passive transport of small molecules into the cell (Iyer et al., 2018; Samsudin et al., 2016). Adhesins, including the high molecular weight BAP protein, contribute to biofilm formation, but their large size and presence of truncated variants pose challenges for vaccine development (De Gregorio et al., 2015; Loehfelm et al., 2008). In contrast, protein Blp1, structurally similar to BAP proteins, aids in biofilm formation and adhesion to human epithelial cells. The C-terminal domain of Blp1 elicits an immune response and protect mice from bacterial infection during active and passive immunizations (Skerniškytė et al., 2019). These insights highlight the potential of the C-terminal domain of Blp1 and OmpA proteins as vaccine candidates against *A. baumannii*.

In our previous work, we demonstrated the use of the tail tube protein gp39 of bacteriophage vB_EcoS_NBD2 (NBD2) as a tubular carrier for the presentation of foreign epitopes (Špakova et al., 2020). Building on this work, the aim of this study is to investigate the immunogenicity of these polytubes in mice and evaluate their ability to elicit IgG response against OmpA foreign protein fragments displayed on the polytubes. By expanding the knowledge about the immunogenicity and efficacy of the polytubes as a carrier for foreign epitope display, our study contributes to the development of a novel immunogenic carrier for antigen presentation and vaccine development.

## 2 Materials and methods

### 2.1 Construction of expression vectors

**Fig. 1:**
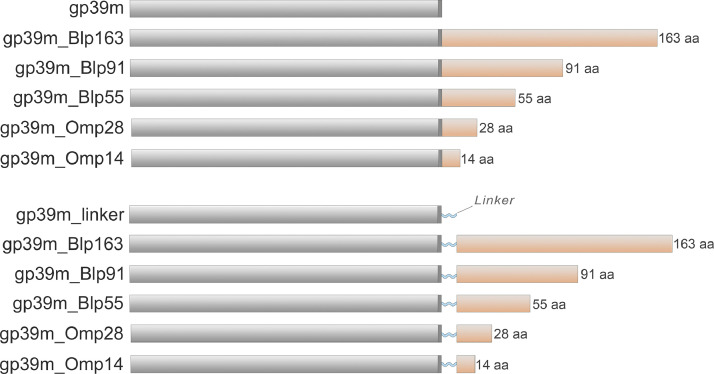
A graphical illustration of the chimeric gp39 proteins. The cloning site inserted into the C-terminus of gp39 is highlighted in a dark grey box, while the (GGGGS)_3x_-linker is represented by a light blue shape. The protein inserts are depicted in light orange boxes, with the Blp1 and OmpA protein fragments labeled according to their length in aa sequences.

To express chimeric proteins, DNA sequences encoding fragments of Blp1 protein (163 amino acids (aa), 91 aa and 55 aa in length) and fragments of OmpA protein (28 aa and 14 aa in length) were amplified from the *A. baumannii* cell lysate (Skerniškytė et al., 2021) using primers and oligonucleotides (Invitrogen, Groningen, The Netherland) listed in Supplementary Table S1. The obtained DNA fragments (Blp163, Blp91, Blp55, Omp28, and Omp14) were cleaved with SmaI and *Bam*HI restriction endonucleases (RE) (Thermo Fisher Scientific Baltics, Vilnius, Lithuania) and inserted into previously constructed pFX7_NBD2_gp39m and pFX7_NBD2_gp39m_linker yeast expression vectors (Špakova et al., 2020). Vectors encoding chimeric proteins with and without the linker (gp39m_linker_Blp163, gp39m_linker_Blp91, gp39m_linker_Blp55, gp39m_linker_Omp28, gp39m_linker_Omp14, gp39m_Blp163, gp39m_Blp91, gp39m_Blp55, gp39m_Omp28, and gp39m_Omp14) were screened in *E. coli* DH5αF’ strain (Department of Eukaryote Gene Engineering, Institute of Biotechnology, Life Sciences Center, Vilnius University) and verified by sequencing (Figs. 1, Supplementary S1).

### 2.2 Heterologous protein synthesis in S. cerevisiae and their purification

Heterologous protein synthesis was carried out in *S. cerevisiae* AH22–214 strain (laboratory strain from the Department of Eukaryote Gene Engineering, Vilnius University). The cultivation of transformed yeast cells and recombinant protein synthesis were performed as previously described by Špakova et al. (2020) with some minor modifications. Briefly, cultivated yeast cells were mechanically disrupted through vortexing with glass beads with the disruption buffer (DB450+Arg: 10 mM Tris–HCl, 450 mM NaCl, 1 mM CaCl_2_, 0.01 % TritonX-100, pH 7.2, 250 mM Arg, 2 mM PMSF). The protein samples were centrifuged at 10,000 × *g* for 40 min at 4 °C (Beckman Coulter Avanti J26 XP Centrifuge, Brea, CA, USA) for the separation of soluble and insoluble proteins. Cell lysate as well as soluble and insoluble protein samples were analyzed by sodium dodecyl sulfate polyacrylamide gel electrophoresis (SDS-PAGE). For the calculations of protein synthesis efficiency in yeast cell lysate samples, the intensities of the desired protein bands were measured with ImageJ 1.50b software (Schneider et al., 2012).

Heterologous proteins gp39, gp39m_linker and gp39m_linker_Omp28 were purified according to the previously described methodology (Špakova et al., 2020; 2019) with minor changes. Briefly, the soluble protein fractions from the yeast cells synthesizing heterologous proteins were transferred onto a 30–40 % (*w/v*) sucrose gradient (10 ml of 40 % and 4 ml of 30 % sucrose solution) in DB150+Arg buffer (10 mM Tris-HCl, 150 mM NaCl, 1 mM CaCl_2_, 0.01 % TritonX-100, pH 7.2, 250 mM Arg) and sedimented by centrifugation at 140,000 × *g* for 2–3 h at 4 °C (Beckman Coulter Optima l-90 K ultracentrifuge). The protein precipitates were suspended and analyzed by SDS-PAGE. If needed, suspended protein pellets were additionally transferred onto a 30–60 % (*w/v*) sucrose gradient (1.5 ml of 60 %, 55 %, 50 %, 45 %, 40 % and 2 ml of 30 % sucrose solution) in DB150+Arg-buffer and spun at 140,000 × *g* for 17–24 h at 4 °C (Beckman Coulter Optima l-90 K ultracentrifuge). Approximately one-milliliter fractions of sucrose solutions were collected and analyzed by SDS-PAGE. Protein fractions having the highest concentration of the target proteins were combined and dialyzed against phosphate buffer (PBS: 0.08 M Na_2_HPO_4_, 0.025 M NaH_2_PO_4_, 0.1 M NaCl, pH 7.4).

### 2.3 SDS-PAGE and western blot analysis

The protein samples were fractionated by SDS-PAGE using 14 % polyacrylamide gels. Then the gels were transferred onto polyvinylidene difluoride (PVDF) blotting membranes (GE Healthcare Life Science, Freiburg, Germany) under semi-dry conditions. The blocking step was carried out in 1 × Roti-Block solution (Carl Roth GmbH and Co, Karlsruhe, Germany) for 1 h at room temperature with shaking. After several washes with Tris-buffered saline (TBS) containing 0.1 % Tween 20 (TBS-T), membranes were incubated per night with gp39 protein-specific polyclonal antibody (2 μg/ml in TBS-T, an in-house produced murine antibody from the Institute of Biotechnology, Vilnius University). After washing with TBS-T, the membranes were incubated for 2 h with HRP-conjugated anti-mouse IgG (1:3000 in TBS-T, BioRad). After several washes with TBS-T and the final wash with TBS, the detection based on the HRP reaction was carried out by adding 4-chloro-1-naphthol and H_2_O_2_ (Fluka, Buchs, Switzerland).

### 2.4 Transmission electron microscopy

Analysis under electron microscopy was carried out as described previously by Špakova et al. (2020).

### 2.5 Immunization of mice and dosage formulations

**Fig. 2:**
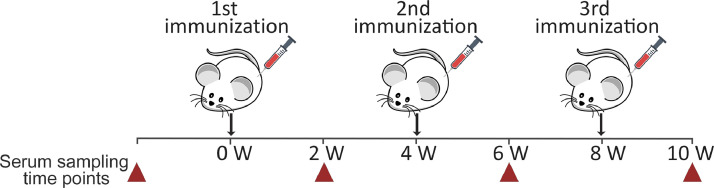
A schematic representation of mice immunizations. Mice were subcutaneously injected three times with the recombinant protein gp39 with or without Freund's adjuvant. The time scale is indicated in weeks. Serum samples were collected with a two-week interval between each immunization. Sampling is indicated by red triangles.

To assess the immunogenicity of polytubes formed by the unmodified protein gp39, female 9-week-old BALB/c mice were used (Department of Biomodels, Institute of Biochemistry, Life Sciences Center, Vilnius University, Lithuania). Two groups of 5 mice were administered subcutaneous immunizations with 50 µg of recombinant protein gp39 in 100 µl of sterile PBS, with or without the adjuvant, at 4-week intervals for a total of 3 immunizations (Fig. 2). The first immunization with adjuvant involved mixing 100 µl of recombinant protein gp39 with an equal volume of complete Freund's adjuvant (Sigma Aldrich, St. Louis, MO, USA). For the second immunization on week 4, incomplete Freund's adjuvant was used. The boost immunization on week 8 was administered without any adjuvant. Serum samples were taken from the tail bleeds from pre-immunized mice as well as two-weeks after each injection as shown in Fig. 2.

To compare the immunogenicity of the Omp28 protein fragment displayed on the polytubes (gp39m_linker_Omp28) with that of Omp28 peptide alone, four protein formulations were used: (1) chimeric protein gp39m_linker_Omp28, (2) protein gp39m_linker mixed with unconjugated Omp28 peptide, (3) Omp28 peptide alone, and (4) unmodified protein gp39 as a control. To ensure equal dosage of formulations, the ratio of protein gp39 to the Omp28 peptide within the chimeric protein gp39m_linker_Omp28 was calculated. The percent of Omp28 peptide relative to the total protein mass of gp39 in the chimeric gp39m_linker_Omp28 (28.93 kDa) was taken as the ratio of Omp28 molecular mass (3 kDa) relative to that of the recombinant protein gp39 monomer (24.36 kDa), as there would be only one copy of Omp28 within each gp39 protein monomer forming the polytubes. Based on the calculations, a 50 µg dose of chimeric protein gp39m_linker_Omp28 was equivalent to 42.1 µg of recombinant protein gp39 and 5.2 µg of Omp28 peptide. The immunization scheme and time points were equivalent to the previously described protocol (Fig. 2). Subcutaneous immunization was administered to four groups of five 9-week-old BALB/c mice (Department of Biomodels) at 4-week intervals with 50 µg of chimeric protein gp39m_linker_Omp28, 42.1 µg of protein gp39m_linker mixed with 5.2 µg of unconjugated Omp28 peptide, 5.2 µg of Omp28 peptide alone, and 42.1 µg of unmodified protein gp39 in 100 µl of sterile PBS. Serum samples were obtained from pre-immunized mice and two weeks after each injection. The protein samples were mixed with sterile PBS to 100 µl volume. The Omp28 peptide was synthesized by GenScript (Piscataway, NJ, USA).

All procedures involving experimental mice were performed in accordance with the Lithuanian and European legislation. The approval to use BALB/c mice for immunizations was obtained from the Lithuanian State Food and Veterinary Agency (permission No. G2–117, issued 11–06–2019).

### 2.6 Indirect ELISA

The specific IgG antibody titers against recombinant gp39, chimeric gp39m_linker_Omp28 and Omp28 were determined by an indirect ELISA. Sucrose-purified proteins gp39 or gp39m_linker_Omp28 were coated onto flat-bottomed microtiter plates (Nerbe Plus GmbH, Winsen/Luhe, Germany) at a concentration of 200 ng per well diluted in coating buffer (50 mM Na_2_CO_3_, pH 9.6) and incubated overnight at 4 °C. The plates were then washed 3 times with PBS-T (PBS with 0.05 % Tween-20 (v/v)) (BioRad) and blocked with 1x Roti-Block solution (Carl Roth GmbH and Co) for 1 h at RT. After washing 3 times with PBS-T, 100 µl of 2-fold serially diluted serum samples in PBS-T were added, followed by an hour of incubation at 37 °C. After washing 5 times with PBS-T, 100 µl of HRP-conjugated anti-mouse IgG (1:3000 in PBS-T, BioRad) were added and incubated for 1 h at 37 °C. After 5 washes with PBS-T, 100 µl of TMB substrate (Clinical Science Products Inc., Mansfield, Mass., USA) was added to each well. The reaction was stopped by adding an equal volume of 10 % H_2_SO_4_ to the each well. The OD values were measured at 450 nm (with the reference OD at 620 nm) using the Multiskan EX plate reader (Franklin, MA, USA).

To determine IgG antibody titers against OmpA peptide, Maxi-Sorp™ microtiter plates (Thermo Fisher, Vilnius, Lithuania) were coated with 250 ng of peptide per well diluted in distilled water and left to dry overnight at 37 °C. The plates were blocked with 2 % bovine serum albumin in PBS for 1 h at RT. Two-fold serially diluted serum samples were added and the plates were incubated for 1 h at RT. Following washing the plates, HRP-conjugated anti-mouse IgG (1:5000 in PBS-T, BioRad) were added. After 1 h of incubation at 37 °C, TMB substrate (Clinical Science Products Inc., Mansfield, Mass., USA) was added and the reactions were stopped by adding 50 µl of 3.5 % H_2_SO_4_ to each well. The OD values were measured at 450 nm (with the reference OD at 620 nm) using plate spectrophotometer (Multiskan GO, Thermo Fisher Scientific, USA).

### 2.7 Statistical analysis

The antibody titers of immunized mice sera were defined as the final serum dilution giving OD value that was 3 standard deviations (SD) above the mean OD for the negative control serum and at least 0.2 (x̅ + 3 SD; *≥* 0.2) (Malm et al., 2014). The antibody titers were tested for normality using *Shapiro-Wilk* test. Since the data was not distributed normally and had small population size (*N* = 5 per group), the nonparametric tests were used. The antibody titers within mice group were compared using *Wilcoxon Signed Rank* test and antibody titers among two mice groups were compared using *Mann-Whitney U* test. Statistical analysis was performed using MS Excel 2016 software and SPSS Statistics (IBM Corp (2015)) version 23.0. Values with *p* < 0.05 were considered statistically significant.

## 3 Results

### 3.1 The immunogenicity of the polytubes formed by the protein gp39

**Fig. 3:**
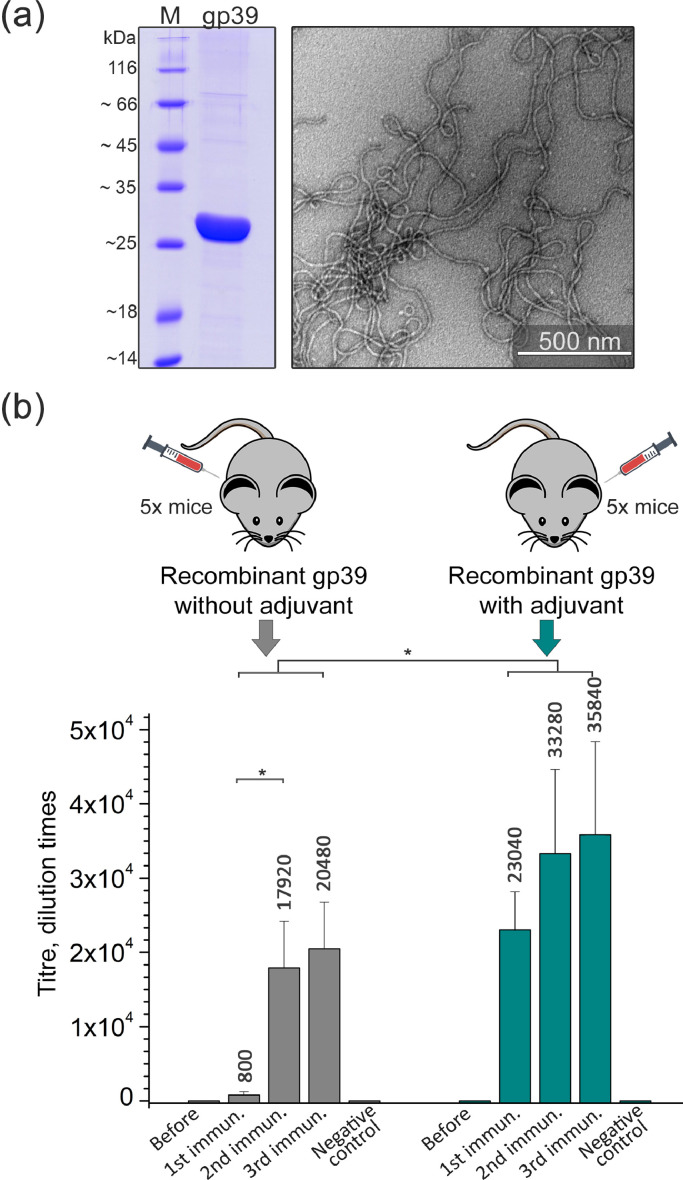
Protein purification, the morphology of the polytubes and their immunogenicity analysis in mice. **(a)** SDS-PAGE and electron micrograph of purified recombinant protein gp39 and the nanotubes it forms. M – Page ruler unstained protein ladder (Thermo Fisher Scientific, Vilnius, Lithuania). **(b)** Two groups of mice (*N* = 5 per group) were immunized with the polytubes formed by the protein gp39 with or without Freund's adjuvant (mouse groups are indicated in different colors). The humoral immune response against gp39 protein was determined by an indirect ELISA. Each histogram represents the mean value of gp39 protein-specific IgG titer within a mouse group, the bars represent mean ± SD. Virus-like particles formed by yeast-derived hamster polyomavirus VP1 protein were used as a negative control. The symbol “*” shows statistically significant difference between the two mice groups (*p* < 0.05). The unmodified, original files can be found in Supplementary Fig. S2.

To determine the immunogenicity of the polytubes, *S. cerevisiae*-produced recombinant tail tube protein gp39 of bacteriophage NBD2 was purified under native conditions. As evidenced by transmission electron microscopy (TEM), the recombinant gp39 self-assembled into well-ordered, long and flexible polytubes (Fig. 3A) (Špakova et al., 2019). The polytube-induced humoral immune response in immunized BALB/c mice was evaluated by testing collected blood samples for the presence of gp39 protein-specific IgG antibodies by an indirect ELISA. Two groups of mice were immunized three times at 4-week intervals and were used to determine the immunogenicity of unmodified polytubes without the use of any adjuvants and to test anti-gp39 humoral immune response using Freund's adjuvant (Fig. 3B, Supplementary Table S2 A).

In the group of mice that received recombinant gp39 without the use of any adjuvant, the initial immunization elicited a gp39-specific antibody response with a mean antibody titer of 1:800. The second immunization with protein gp39 statistically significantly increased gp39-specific antibody response (*p* = 0.046) giving a mean antibody titer of 1:17,920. After the third immunization with the gp39 protein in the absence of any adjuvant, a statistically insignificant rise in the mean antibody titer to 1:20,480 was observed (*p* = 1.0). In the second mouse group immunized with protein gp39 emulsified with Freund's adjuvant, the first immunization resulted in a mean antibody titer of 1:23,040. The second immunization resulted in a statistically insignificant antibody response specified by the mean titer of 1:33,280 (*p* = 0.34). The third immunization with gp39 resulted in antibody titer 1:35,840, which did not differ significantly from the immune response measured from the second immunization (*p* = 1.0) (Fig. 3B, Supplementary Table S2 A).

Comparing gp39-specific antibody titers between two mice groups with or without the use of adjuvant, Freund's adjuvant statistically significantly increased the gp39-specific antibody titers compared with the antibody titers where only protein gp39 was used for mouse immunizations (*p* = 0.001). The specificity of the antiserum raised against gp39 protein was confirmed using yeast-produced hamster polyomavirus VP1 protein (Sasnauskas et al., 1999) as a negative control. Also, no gp39-specific antibodies were detected in the blood samples collected from the pre-immunized mice (Fig. 3B).

### 3.2 Bioinformatics analysis of full-length gp39 protein and protein fragments from Blp1 and OmpA

**Fig. 4:**
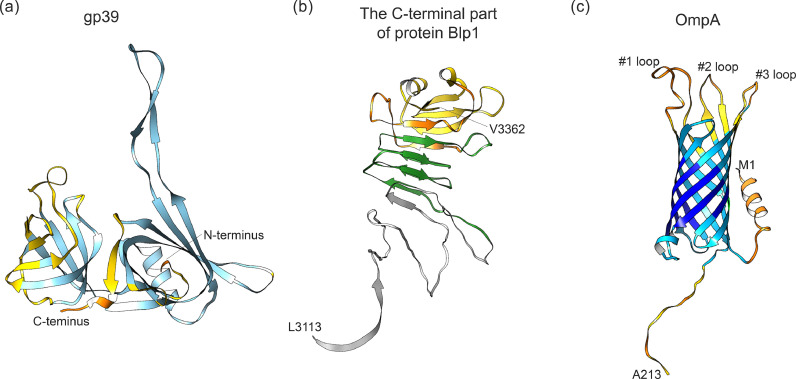
3D models of protein gp39, C-terminal part of protein Blp1 and protein OmpA. **(a)** A putative model of the gp39 monomer. **(b)** The structure of the C-terminus of protein Blp1, spanning 250 aa residues. Within this structure, a fragment referred to as "Blp55″ is depicted in yellow, "Blp91″ is shown in a combination of yellow and orange colors, while yellow, orange, and green colors represent the "Blp163″ fragment. **(c)** The structure of protein OmpA. The colors assigned to gp39 and OmpA correspond to the pLDDT scores across the predicted structures, where dark blue (scores > 90 %) suggests very confident prediction of the backbone, light blue (between 70 and 90 %) suggests confident prediction of the backbone, yellow (between 50 and 70 %) indicates low prediction confidence, and orange (below 50 %) denotes a very low prediction level. Structure prediction was conducted using the AlphaFold server (Varadi et al., 2021), while structure visualization was performed using UCSF Chimera v1.17 (Pettersen et al., 2004).

To extract further insights into the structure of the tail tube protein gp39, the HHpred protein homology server (Zimmerman et al., 2018) was employed. Analysis based on the aa sequence of gp39 revealed that aa residues 101–223 exhibit the closest structural similarity to the tail tube protein gpV of phage lambda (PDB: 7T2E), with a probability of 99.58 % (E-value, 2.1e-13). Subsequently, to determine the putative 3D structure of gp39, the complete protein sequence was submitted to the AlphaFold server (Varadi et al., 2021), and the highest-ranked model was selected for further investigation. The confidence levels within the putative model of gp39 were indicated by the predicted local distance difference test (pLDDT) scores (Fig. 4A). While the predicted confidence levels spanned between 70% and 90 % throughout the entirety of gp39, lower confidence in structure prediction was observed within amino acid residues 75–90, particularly at the terminal regions of both the N- and C-terminal ends of gp39 (specifically residues 1–9 and 210–223, with pLDDT scores < 50 %). Our data reveal that the N-terminal segment of protein gp39 comprises β-sheets, α-helices, and an elongated loop similar to the TE-loop observed in the homologous tail tube protein gpV of phage lambda (Fig. 4A) (Campbell et al., 2020). However, a possible difference between these structures lies in the organization of their C-terminal ends. While the Ig-like fold is present in gpV and in the majority of other tailed bacteriophages (Linares et al., 2020), the C-terminal part of gp39 is unlikely to possess this domain (Fig. 4A).

Potential Blp1 and OmpA protein fragments for the insertion into the C-terminus of gp39 were selected based on structural analysis. The 3D structure of the C-terminal end of Blp1, spanning 250 amino acids, was generated using AlphaFold. The highest-ranked model, bearing 100 % sequence identity to the Type I secretion C-terminal target domain-containing protein of *A. baumannii* (UNIPROT: A0A6I4IHW6), was chosen for further analysis. Considering that the insertion of multiple β-sheets could potentially interfere the self-assembly into polytubes of protein gp39, smaller Blp1 protein fragments were selected. These fragments include one comprising 163 aa (encompassing Blp1 residues 3200–3363, denoted as Blp163), another of 91 aa (residues 3272–3363, referred to as Blp91), and a third of 55 aa (residues 3308–3363, named Blp55) (Fig. 4B). Analysis by AlphaFold revealed a 100 % identity between the complete sequence of the OmpA protein and the OmpA-like protein of *A. baumannii* (strain AYE, UNIPROT: B0V4Y8). According to this model, OmpA adopts a β-barrel structure with three loops, predominantly oriented towards the exterior of the cell. As loop #3 exhibited the least conservation among *A. baumannii* variants (Bosák et al., 2016; unpublished data), and considering structural aspects and the feasibility of displaying foreign protein fragments on the polytubes, only loops #1 and #2 were selected for further investigation. Two regions were chosen for insertion into the C-terminus of gp39: one spanning 28 aa (encompassing OmpA residues 32–59, denoted as loop #1), and another spanning 14 aa (residues 83–96, referred to as loop #2), named Omp28 and Omp14, respectively (Fig. 4C).

### 3.3 Production of chimeric proteins and characterization of the polytubes

We have previously generated chimeric polytubes formed by the protein gp39 with C-terminal insertions spanning from 6 aa to 238 aa and demonstrated their different abilities to self-assemble into polytubes (Špakova et al., 2020). To minimize the impact of Blp1 and OmpA protein fragment insertions into the C-terminus of gp39m on polytube formation, additional protein variant having C-terminal insertion of glycine-serine linker (gp39m_linker) was used for the generation of chimeric proteins.

**Fig. 5:**
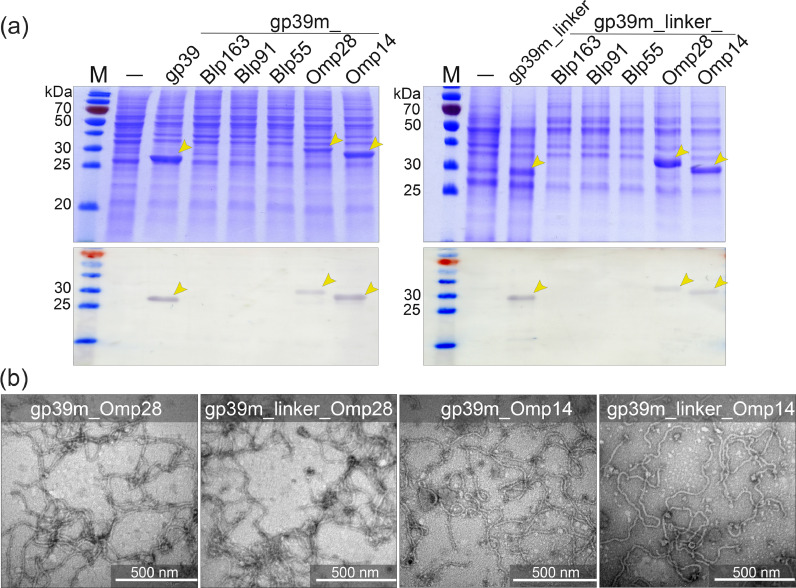
Analysis of chimeric protein gp39 variants with inserted Blp1 or OmpA protein fragments. **(a)** SDS-PAGE and Western blot analysis of the yeast-produced chimeric gp39 proteins. M *–* Page Ruler™ Prestained protein ladder (Thermo Fisher Scientific Baltics, Lithuania); „–“ indicates the lysate of yeast cells transformed with an “empty” vector pFX7. The lysates of yeast cells synthesizing target proteins are shown. For Western blot analysis, gp39 protein-specific polyclonal antibodies were used (VU IBT). Abbreviation “link” means glycine-serine linker. **(b)** Electron micrographs of the nanotubes. The scale bars are indicated. The unmodified, original files can be found in Supplementary Fig. S3.

Following chimeric protein synthesis in *S. cerevisiae*, the insertions of protein fragments Omp14 and Omp28 into the C-terminus of gp39m and gp39m_linker were well-tolerated (Fig. 5). While the amount of chimeric proteins gp39m_Omp14 and gp39m_linker_Omp14 in yeast cell lysate samples was similar to that of the unmodified protein gp39, the synthesis of chimeric proteins with Omp28 fragment demonstrated higher efficiency when employing a serine-glycine linker (gp39m_linker_Omp28) (Fig. 5A). Conversely, the insertion of Blp1 protein fragments (Blp163, Blp91, and Blp55) into gp39m and gp39m_linker had a profound impact on synthesis efficiency, as these chimeric proteins were not detectable in the yeast cell lysates (Fig. 5A).

To assess the self-assembly properties of the purified chimeric proteins (gp39m_Omp14, gp39m_linker_Omp14, gp39m_Omp28, and gp39m_linker_Omp28), TEM was employed. TEM analysis revealed that the insertion of Omp28 and Omp14 fragments at the C-terminus of gp39m and gp39m_linker was well-tolerated, and the morphology of the resulting polytubes did not depend on the use of linker. The chimeric proteins self-assembled into well-ordered, flexible polytubes, displaying a morphology similar to that of the polytubes formed by the unmodified protein gp39 with a width ranging from 12nm to 14 nm and a length varying from 0.1 µm to > 1.2 µm (Fig. 5B).

### 3.4 IgG response against ompa protein fragments displayed on the polytubes

**Fig. 6:**
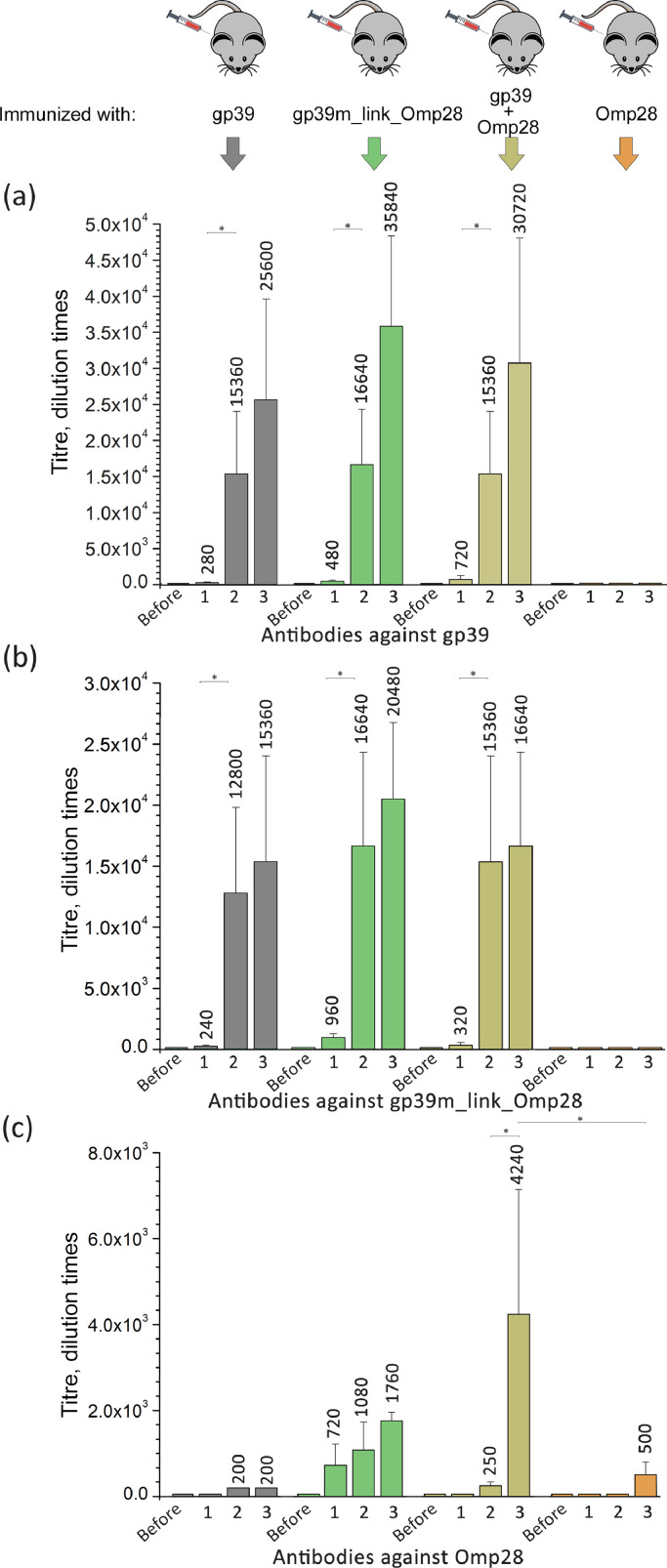
Immunogenicity analysis of Omp28 peptide when presented on polytubes versus its isolated form. Four groups of five mice were immunized three times with protein gp39 (shown in grey), chimeric gp39m_link_Omp28 (green), protein gp39 formulated together with the unconjugated peptide Omp28 (light brown) and with Omp28 peptide alone (orange). IgG antibody titers against **(a)** protein gp39, **(b)** chimeric gp39m_link_Omp28, and **(c)** peptide Omp28 were determined by an indirect ELISA. Data is obtained from two identical and independent experiments. Every histogram illustrates the average of protein or peptide-specific IgG antibody titer within each group of mice, the bars represent mean ± SD. Statistically significant differences between mice groups (*p* < 0.05) are shown in “*” as determined by Wilcoxon Signed Rank or Mann-Whitney U tests.

In order to assess whether gp39-derived polytubes were able to induce the production of antibodies against the foreign Omp28 peptide incorporated in its sequence, we conducted two identical and independent ELISA experiments to assess and compare the humoral immune responses in mice against the carrier protein gp39, the chimeric protein gp39m_link_Omp28, and the epitope Omp28. For this, four mouse groups (*N* = 5) were immunized with either the polytubes formed by the unmodified gp39, or chimeric protein gp39m_link_Omp28, or protein gp39 formulated together with the unconjugated Omp28 or with Omp28 peptide alone (Fig. 6).

Mouse immunizations with gp39, gp39m_link_Omp28 and with protein gp39 formulated together with unconjugated Omp28 resulted in similar IgG antibody titers against proteins gp39 or gp39m_link_Omp28 with no significant differences among three mouse groups (Fig. 6A, B, Supplementary Table S2 B). The primary immunizations of mice resulted in the mean gp39- or gp39m_link_Omp28-protein specific antibody titers up to 1:960. Following the second immunizations, the mean antibody titers against protein gp39 or gp39m_link_Omp28 statistically significantly increased up to 1:16,640. After the third immunizations, no statistically significant differences in mean antibody titers against gp39 or gp39m_link_Omp28 were obtained between mouse groups (antibody titers ranged from 1:15,360 to 1:35,840). As expected, no gp39- or gp39m_link_Omp28-specific antibodies were detected in the blood samples from pre-immunized mice and in the mouse group immunized with Omp28 peptide alone (Fig. 6A, Supplementary Table S2 B).

Immunizations of mice with chimeric gp39m_link_Omp28 resulted in the production of Omp28-specific IgG antibodies. Following the primary immunization, the mean antibody titer against Omp28 reached 1:720. Subsequent immunizations led to an increase in mean Omp28-specific antibody titers, ranging from 1:1080 to 1:1760, with no statistically significant variations observed between immunizations (Fig. 6C, Supplementary Table S2 B). Notably, immunizations of mice with gp39 formulated together with the unconjugated Omp28 peptide resulted in the highest antibody titer against Omp28. While the levels of antibodies against Omp28 after the first and the second immunizations were nearly undetectable (up to 1:250), the third immunization significantly increased Omp28-specific IgG antibody titers to 1:4240 (*p* = 0.043). As expected, immunization with Omp28 peptide alone failed to elicit a humoral immune response. Only after the third immunization with this peptide, a low Omp28-specific antibody titer of 1:500 was observed. Furthermore, no Omp28-specific IgG titers were detected in mice immunized with gp39 alone. Non-specific antibody titers of 1:200 were observed after the second and the third immunizations (Fig. 6C, Supplementary Table S2 B). The specificity of all of the tested sera was confirmed by analyzing yeast-produced hamster polyomavirus VP1 protein (Sasnauskas et al., 1999) as a negative control (data not shown), the p values are given in Supplementary Table S2.

## 4 Discussion

This study shows the immunogenicity of flexible polytubes formed by the bacteriophage tail tube protein gp39, spanning from 0.1 µm to over 3.95 µm in length. The administration of gp39-based polytubes in mice induced a T cell-dependent B cell response, leading to the production of gp39 protein-specific IgG antibodies. While the immunogenicity of icosahedral virus-like particles has been extensively explored (Kheirvari et al., 2023), the immunogenicity of extremely long and flexible polytubes remains less studied. Only recently more attention has been directed towards the immunogenicity of flexuous virus-based polytubes mostly derived from viruses as papaya mosaic virus, turnip mosaic virus, potato virus X and Y, or even elongated nanoparticles from cowpea chlorotic mottle virus (Carignan et al., 2015; Ogrina et al., 2022; Savard et al., 2011; Truchado et al., 2023; Zinkhan et al., 2021). Our study expands the potential applications of flexible and remarkably long polytubes formed by the gp39 protein, as we demonstrate their immunogenicity even in the absence of adjuvants.

As expected, the use of recombinant gp39 protein emulsified with an adjuvant significantly enhanced the titers of gp39-specific antibodies compared to the use of gp39 protein alone. The ability of adjuvants to boost protein-specific antibody titers has been demonstrated in other studies with various virus-like particles (Gedvilaite et al., 2004; Kalnciema et al., 2011; Spohn et al., 2010) and adjuvants are widely employed in licensed vaccines. While the third immunization with gp39 in both groups of mice did not yield a statistically significant increase in antibody titers compared to the second immunization, these findings suggest that an efficient IgG antibody response can be achieved after two immunizations with gp39 protein, with or without the use of an adjuvant. Notably, some virus-like particle-based licensed vaccines require three doses to elicit an effective immune response and ensure adequate protection against viruses (FDA, 2019; Merck, 2021a, 2021b). Although further research is needed, the polytubes formed by the gp39 hold a promise as vaccine candidate, potentially offering the advantage of achieving optimal efficacy with just two doses.

Like for many other filamentous viruses, the crystal structure of polytubes formed by the NBD2 phage tail tube protein gp39 has not yet been determined. This lack of structural information poses challenges in identifying surface regions of gp39 that are suitable for the insertion of foreign protein fragments. Bioinformatics analysis revealed a topological similarity between the protein gp39 and the tail tube protein gpV of phage lambda. Both proteins share common features at their N-terminal ends, including β-sheets, α-helix, and a long loop known as the TE-loop in gpV (Campbell et al., 2020). However, a possible difference between these structures lies in the organization of their C-terminal ends. While the Ig-like fold is present in gpV, the C-terminal part of gp39 is unlikely to possess this domain. This observation aligns with the fact that not all tailed phages exhibit the Ig-like domain (Linares et al., 2020). Consequently, the tolerance of gp39 for the insertion of foreign protein fragments at its C-terminus can be attributed to these structural distinctions and the fact, that aa deletion from the C-terminus of gp39 negatively affected protein synthesis efficiency and self-assembly properties (Špakova et al., 2020).

Recognizing that individual proteins and peptides are often poorly immunogenic, the utilization of viral nanostructures has proven to be a highly effective approach to enhance their immunogenicity (Carignan et al., 2015; Denis et al., 2007; Jaber Hossain et al., 2011; Peabody et al., 2009). In our previous work, we characterized the polytubes formed by the tail tube protein gp39 as a carrier for displaying foreign epitopes, specifically identifying the C-terminal region suitable for the insertion of foreign protein fragments. Notably, the C-terminal part of gp39 tolerated the insertions of 6–238 aa-long fragments on the exterior surface of the polytubes, but the aa composition and the insert size influenced either synthesis efficiency of chimeric proteins in yeast or their ability to self-assemble into typical polytubes (Špakova et al., 2020).

Building upon these findings, in this study we selected two proteins, OmpA and Blp1, derived from *A. baumannii,* as promising candidates for vaccine development (Badmasti et al., 2015; Luo et al., 2012; Skerniškytė et al., 2021; 2019). To insert protein fragments to the C-terminus of gp39 while minimizing disruption of the gp39 tertiary structure, we chose aa sequences corresponding to the loop regions of OmpA protein and selectively shortened protein fragments of Blp1, which consisted of multiple β-sheets. Notably, the insertions of 28- and 14-aa-long fragments from OmpA protein into the C-terminus of gp39 were well-tolerated and resulted in the formation of flexible and long polytubes. However, the insertion of 163-, 91-, and 55-aa-long fragments from Blp1 protein had a profound impact on protein synthesis efficiency, leading to the absence of polytubes. Presumably, foreign protein inserts should not form β-sheets to prevent adverse effects on the tertiary structure of the scaffold protein gp39. To facilitate the effective presentation of Blp1 protein fragments and other “large” proteins on these polytubes, longer linkers could be employed as spacers allowing proteins to have a correct tertiary fold without disturbing self-assembly properties of the scaffold protein (Kalnciema et al., 2011; Urakami et al., 2017; Zvirbliene et al., 2006). Furthermore, the reduction of steric hindrance between the scaffold protein gp39 and foreign proteins could be achieved through the synthesis of mosaic polytubes, which would consist of unmodified gp39 subunits along with chimeric proteins with inserted foreign protein fragments. Previous studies have demonstrated the successful implementation of this strategy with other viral nanostructures emphasizing its effectiveness (Brown et al., 2009; Röder et al., 2017), but the display of larger and structurally more complex proteins is still a limiting factor for the majority of viral nanostructures.

This study represents the first comparative analysis of the immunogenicity of protein fragments either in their monomeric form and displayed on bacteriophage-originated polytubes. As anticipated, the monomeric Omp28 protein fragment was non-immunogenic in mice, whereas chimeric polytubes, harboring C-terminal insertion of Omp28 protein fragment, induced the production of Omp28 peptide-specific IgG antibodies, even in the absence of adjuvants. The structural features of these polytubes facilitate the presentation of epitopes in a repetitive and highly ordered manner, thereby augmenting the immunogenicity for non-immunogenic Omp28 fragments. In a previous investigation, we elucidated the surface localization of foreign protein fragments when inserted into the C-terminus of the scaffold protein gp39 (Špakova et al., 2020). This is important, since the accessibility of epitopes to the immune system components and the multimerization are crucial prerequisites for eliciting a robust humoral immune response, as supported by numerous studies (Czarnota et al., 2016; Denis et al., 2007).

As expected, subcutaneous immunizations with chimeric polytubes (gp39m_link_Omp28 protein) elicited a humoral immune response against Omp28. Interestingly, the third immunization with gp39-formed polytubes together with the unconjugated Omp28 significantly elevated antibody titers against Omp28 peptide. While further analysis is required, our data suggests that gp39-formed polytubes could potentially be used as an adjuvant, as indicated by the robust humoral immune response observed against the Omp28 peptide after the third round of immunizations in mice. This potential feature of gp39-formed polytubes, characterized by their self-adjuvating properties, may eliminate the need to use error-prone genetic engineering steps for the insertion and display of foreign peptides on the surface of polytubes.

In previous studies, viral nanostructures have been predominantly used to display non-immunogenic protein fragments to enhance their immunogenicity (Ghorbani et al., 2020; Guo et al., 2019; Mohsen et al., 2020). However, there is a limited number of research demonstrating the adjuvant properties of viral nanostructures for improving the immunogenicity of monomeric protein fragments. For instance, VLPs composed of papaya mosaic virus coat proteins were shown to be used as a vaccine carrier and adjuvant, improving the protection provided by the trivalent inactivated flu vaccine (Carignan et al., 2015; Denis et al., 2008; Savard et al., 2011). Similarly, icosahedral simian virus 40-based VLPs have exhibited intrinsic adjuvant properties when used together with the influenza vaccine (Kawano et al., 2014). Notably, self-adjuvanting properties have mainly been studied in icosahedral VLPs and shorter virus-based polytubes. In this study, we introduce novel bacteriophage-originated, extremely-long polytubes, showcasing their versatility as both immunogenic antigen carrier and potential adjuvant in vaccine development.

## 5 Conclusions

In conclusion, our study demonstrates the versatility and immunogenicity of gp39-formed polytubes, establishing them as promising vaccine candidates. The successful insertion of OmpA protein fragments into the C-terminus of gp39, without compromising polytube morphology, further supports their suitability as a carrier for antigen presentation. However, caution should be exercised when inserting foreign protein fragments that form β-sheets, as this may affect the fold of protein gp39 and its ability to self-assemble. Notably, even without the use of adjuvants, the polytubes exhibit strong immunogenic properties and induce humoral immune response against the foreign epitope Omp28, suggesting their potential use in vaccine development.

Overall, our findings highlight the potential of gp39-formed polytubes as an immunogenic platform to display target protein fragments. While further analysis is needed, the current data suggests that gp39-formed polytubes could potentially be used as an adjuvant in combination with unconjugated antigens. The self-adjuvant property of polytubes could be regarded as an outstanding advantage allowing their straightforward use in vaccine formulations while obviating the necessity for error-prone genetic engineering steps and eliminating the need for potentially toxic adjuvants.

## Funding

This research was funded by a grant (No. S-SEN-20–1) from the Research Council of Lithuania.

## CRediT authorship contribution statement

**Aliona Avižinienė:** Writing – original draft, Visualization, Methodology, Investigation, Data curation, Conceptualization. **Indrė Dalgėdienė:** Writing – review & editing, Methodology, Investigation, Conceptualization. **Julija Armalytė:** Resources, Funding acquisition, Conceptualization. **Rasa Petraitytė-Burneikienė:** Writing – review & editing, Methodology, Investigation, Conceptualization.

## Declaration of competing interest

The authors declare that they have no known competing financial interests or personal relationships that could have appeared to influence the work reported in this paper.

## Appendix Supplementary materials

**Supplementary Materials:** Supplementary Table S1: Oligonucleotides used in this work; Supplementary Fig. S1: Expression vectors used for the synthesis of chimeric proteins in *S. cerevisiae*; Supplementary Fig. S2: Analysis of purified recombinant protein gp39 **(a)** by SDS-PAGE and **(b)** electron microscopy; Supplementary Fig. S3: Characterization of recombinant protein gp39 variants; Supplementary Table S2: The *p*-values of conducted immunological experiments in mice. Image, application 1

## References

[bib0001] Badmasti F., Ajdary S., Bouzari S., Fooladi A.A.I., Shahcheraghi F., Siadat S.D. (2015). Immunological evaluation of OMV(PagL)+Bap(1-487aa) and AbOmpA(8-346aa)+Bap(1-487aa) as vaccine candidates against Acinetobacter baumannii sepsis infection. Mol. Immunol..

[bib0002] Bosák J., Micenková L., Doležalová M., Šmajs D. (2016). Colicins U and Y inhibit growth of Escherichia coli strains via recognition of conserved OmpA extracellular loop 1. Int. J. Med. Microbiol..

[bib0003] Brown S.D., Fiedler J.D., Finn M.G. (2009). Assembly of hybrid bacteriophage Qbeta virus-like particles. Biochemistry.

[bib0004] Campbell P.L., Duda R.L., Nassur J., Conway J.F., Huet A. (2020). Mobile loops and electrostatic interactions maintain the flexible tail tube of bacteriophage Lambda. J. Mol. Biol..

[bib0005] Carignan D., Thérien A., Rioux G., Paquet G., Gagné M.È.L., Bolduc M., Savard P., Leclerc D. (2015). Engineering of the PapMV vaccine platform with a shortened M2e peptide leads to an effective one dose influenza vaccine. Vaccine.

[bib0006] Chen N., Zhou M., Dong X., Qu J., Gong F., Han Y., Qiu Y., Wang J., Liu Y., Wei Y., Xia J., Yu T., Zhang X., Zhang L. (2020). Epidemiological and clinical characteristics of 99 cases of 2019 novel coronavirus pneumonia in Wuhan, China: a descriptive study. Lancet.

[bib0007] Chen T.H., Hu C.C., Lee C.W., Feng Y.M., Lin N.S., Hsu Y.H. (2021). Stable display of artificially long foreign antigens on chimeric bamboo mosaic virus particles. Viruses.

[bib0008] IBM Corp (2015).

[bib0009] Czarnota A., Tyborowska J., Peszyńska-Sularz G., Gromadzka B., Bieńkowska-Szewczyk K., Grzyb K. (2016). Immunogenicity of Leishmania-derived hepatitis B small surface antigen particles exposing highly conserved E2 epitope of hepatitis C virus. Microb. Cell Fact..

[bib0010] De Gregorio E., Del Franco M., Martinucci M., Roscetto E., Zarrilli R., Di Nocera P.P. (2015). Biofilm-associated proteins: news from Acinetobacter. BMC Genom..

[bib0011] Denis J., Acosta-Ramirez E., Zhao Y., Hamelin M.E., Koukavica I., Baz M., Abed Y., Savard C., Pare C., Lopez Macias C., Boivin G., Leclerc D. (2008). Development of a universal influenza A vaccine based on the M2e peptide fused to the papaya mosaic virus (PapMV) vaccine platform. Vaccine.

[bib0012] Denis J., Majeau N., Acosta-Ramirez E., Savard C., Bedard M.C., Simard S., Lecours K., Bolduc M., Pare C., Willems B., Shoukry N., Tessier P., Lacasse P., Lamarre A., Lapointe R., Lopez Macias C., Leclerc D. (2007). Immunogenicity of papaya mosaic virus-like particles fused to a hepatitis C virus epitope: evidence for the critical function of multimerization. Virology.

[bib0013] Fan Y.C., Chiu H.C., Chen L.K., Chang G.J.J., Chiou S.S. (2015). Formalin inactivation of Japanese encephalitis virus vaccine alters the antigenicity and immunogenicity of a neutralization epitope in envelope protein domain III. PLoS Negl. Trop. Dis..

[bib0014] FDA, 2019. ENGERIX-B. Available at: https://www.fda.gov/vaccines-blood-biologics/vaccines/engerix-b. Accessed March 15, 2023.

[bib0015] Frietze K.M., Peabody D.S., Chackerian B. (2016). Engineering virus-like particles as vaccine platforms. Curr. Opin. Virol..

[bib70] Gedvilaite A., Zvirbliene A., Staniulis J., Sasnauskas K., Krüger D.H., Ulrich R. (2004). Segments of Puumala hantavirus nucleocapsid protein inserted into chimeric polyomavirus-derived virus-like particles induce a strong immune response in mice. Viral Immunol..

[bib0016] Ghorbani A., Zare F., Sazegari S., Afsharifar A., Eskandari M.H., Pormohammad A. (2020). Development of a novel platform of virus-like particle (VLP)-based vaccine against COVID-19 by exposing epitopes: an immunoinformatics approach. New Microbes New Infect..

[bib0017] Govasli M.L., Diaz Y., Puntervoll P. (2019). Virus-like particle-display of the enterotoxigenic Escherichia coli heat-stable toxoid STh-A14T elicits neutralizing antibodies in mice. Vaccine.

[bib0018] Guo J., Zhou A., Sun X., Sha W., Ai K., Pan G., Zhou C., Zhou H., Cong H., He S. (2019). Immunogenicity of a virus-like-particle vaccine containing multiple antigenic epitopes of toxoplasma gondii against acute and chronic toxoplasmosis in mice. Front. Immunol..

[bib0019] Hawkes N. (2015). European medicines agency approves first malaria vaccine. BMJ.

[bib0020] Huang X., Wang X., Zhang J., Xia N., Zhao Q. (2017). Escherichia coli-derived virus-like particles in vaccine development. Vaccines.

[bib0021] Iyer R., Moussa S.H., Durand-Réville T.F., Tommasi R., Miller A. (2018). Acinetobacter baumannii OmpA is a selective antibiotic permeant porin. ACS Infect. Dis..

[bib0022] Jaber Hossain M., Bourgeois M., Quan F.S., Lipatov A.S., Song J.M., Chen L.M., Compans R.W., York I., Kang S.M., Donis R.O. (2011). Virus-like particle vaccine containing hemagglutinin confers protection against 2009 H1N1 pandemic influenza. Clin. Vaccine Immunol..

[bib0023] Jochmus I., Schäfer K., Faath S., Müller M., Gissmann L. (1999). Chimeric virus-like particles of the human papillomavirus type 16 (HPV 16) as a prophylactic and therapeutic vaccine. Arch. Med. Res..

[bib0024] Kalnciema I., Skrastina D., Ose V., Pumpens P., Zeltins A. (2011). Potato virus Y-like particles as a new carrier for the presentation of foreign protein stretches. Mol. Biotechnol..

[bib0025] Karpenko L.I., Ivanisenko V.A., Pika I.A., Chikaev N.A., Eroshkin A.M., Veremeiko T.A., Ilyichev A.A. (2000). Insertion of foreign epitopes in HBcAg: how to make the chimeric particle assemble. Amino Acids.

[bib0026] Kawano M., Morikawa K., Suda T., Ohno N., Matsushita S., Akatsuka T., Handa H., Matsui M. (2014). Chimeric SV40 virus-like particles induce specific cytotoxicity and protective immunity against influenza A virus without the need of adjuvants. Virology.

[bib0027] Kheirvari M., Liu H., Tumban E. (2023). Virus-like particle vaccines and platforms for vaccine development. Viruses.

[bib0028] Kirnbauer R., Booy F., Cheng N., Lowy D.R., Schiller J.T. (1992). Papillomavirus L1 major capsid protein self-assembles into virus-like particles that are highly immunogenic. Proc. Natl. Acad. Sci. U. S. A..

[bib0029] Lai C.C., Wang C.Y., Hsueh P.R. (2020). Co-infections among patients with COVID-19: the need for combination therapy with non-anti-SARS-CoV-2 agents?. J. Microbiol. Immunol. Infect..

[bib0030] Lau Y.T., Tan H.S. (2023). Acinetobacter baumannii subunit vaccines: recent progress and challenges. Crit. Rev. Microbiol..

[bib0031] Lawatscheck R., Aleksaite E., Schenk J.A., Micheel B., Jandrig B., Holland G., Sasnauskas K., Gedvilaite A., Ulrich R.G. (2007). Chimeric polyomavirus-derived virus-like particles: the immunogenicity of an inserted peptide applied without adjuvant to mice depends on its insertion site and its flanking linker sequence. Viral Immunol.

[bib0032] Linares R., Arnaud C.A., Degroux S., Schoehn G., Breyton C. (2020). Structure, function and assembly of the long, flexible tail of siphophages. Curr. Opin. Virol..

[bib0033] Loehfelm T.W., Luke N.R., Campagnari A.A. (2008). Identification and characterization of an Acinetobacter baumannii biofilm-associated protein. J. Bacteriol..

[bib0034] Luo G., Lin L., Ibrahim A.S., Baquir B., Pantapalangkoor P., Bonomo R.A., Doi Y., Adams M.D., Russo T.A., Spellberg B. (2012). Active and passive immunization protects against lethal, extreme drug resistant-Acinetobacter baumannii infection. PLoS One.

[bib0035] Mallajosyula J.K., Hiatt E., Hume S., Johnson A., Jeevan T., Chikwamba R., Pogue G.P., Bratcher B., Haydon H., Webby R.J., McCormick A.A. (2014). Single-dose monomeric HA subunit vaccine generates full protection from influenza challenge. Hum. Vaccines Immunother..

[bib0036] Malm M., Uusi-Kerttula H., Vesikari T., Blazevic V. (2014). High serum levels of norovirus genotype-specific blocking antibodies correlate with protection from infection in children. J. Infect. Dis..

[bib0037] Merck, 2021a. Dosage and administration for GARDASIL 9. Available at: https://www.merckvaccines.com/gardasil9/dosingadministration/. Accessed September 20, 2023.

[bib0038] Merck, 2021b. Dosage and administration for RECOMBIVAX HB® [Hepatitis B vaccine (Recombinant)]. Available at: https://www.merckvaccines.com/recombivaxhb/dosageadministration/. Accessed September 20, 2023.

[bib0039] Mohsen M.O., Augusto G., Bachmann M.F. (2020). The 3Ds in virus-like particle based-vaccines: “design, delivery and dynamics. Immunol. Rev..

[bib0040] Ogrina A., Skrastina D., Balke I., Kalnciema I., Jansons J., Bachmann M.F., Zeltins A. (2022). Comparison of bacterial expression systems based on potato virus Y-like particles for vaccine generation. Vaccines.

[bib0041] Peabody D.S., Manifold-Wheeler B., Medford A., Jordan S.K., do Carmo Caldeira J., Chackerian B. (2009). Immunogenic display of diverse peptides on virus-like particles of RNA phage MS2. J. Mol. Biol..

[bib0042] Pettersen E.F., Goddard T.D., Huang C.C., Couch G.S., Greenblatt D.M., Meng E.C., Ferrin T.E. (2004). UCSF Chimera - a visualization system for exploratory research and analysis. J. Comput. Chem..

[bib0043] Rawson T.M., Moore L.S.P., Zhu N., Ranganathan N., Skolimowska K., Gilchrist M., Satta G., Cooke G., Holmes A. (2020). Bacterial and fungal coinfection in individuals with Coronavirus: a rapid review to support COVID-19 antimicrobial prescribing. Clin. Infect. Dis..

[bib0045] Röder J., Fischer R., Commandeur U. (2017). Adoption of the 2A ribosomal skip principle to tobacco mosaic virus for peptide display. Front. Plant Sci..

[bib0046] Samsudin F., Ortiz-Suarez M.L., Piggot T.J., Bond P.J., Khalid S. (2016). OmpA: a flexible clamp for bacterial cell wall attachment. Structure.

[bib71] Sasnauskas K., Buzaite O., Vogel F., Jandrig B., Razanskas R., Staniulis J., Scherneck S., Krüger D.H., Ulrich R. (1999). Yeast cells allow high-level expression and formation of polyomavirus-like particles. Biol. Chem..

[bib0047] Savard C., Guérin A., Drouin K., Bolduc M., Laliberté-Gagné M.E., Dumas M.C., Majeau N., Leclerc D. (2011). Improvement of the trivalent inactivated flu vaccine using PapMV nanoparticles. PLoS One.

[bib0048] Schlick T.L., Ding Z., Kovacs E.W., Francis M.B. (2005). Dual-surface modification of the tobacco mosaic virus. J. Am. Chem. Soc..

[bib0049] Schneider C.A., Rasband W.S., Eliceiri K.W. (2012). NIH Image to ImageJ: 25 years of image analysis. Nat. Methods.

[bib0050] Skerniškytė J., Karazijaitė E., Deschamps J., Krasauskas R., Armalytė J., Briandet R., Sužiedėlienė E. (2019). Blp1 protein shows virulence-associated features and elicits protective immunity to Acinetobacter baumannii infection. BMC Microbiol..

[bib0051] Skerniškytė J., Karazijaitė E., Lučiūnaitė A., Sužiedėlienė E. (2021). OmpA protein-deficient Acinetobacter baumannii outer membrane vesicles trigger reduced inflammatory response. Pathogens.

[bib0052] Špakova A., Dalgėdienė I., Insodaitė R., Sasnauskienė A., Žvirblienė A., Petraitytė-Burneikienė R. (2020). vB_EcoS_NBD2 bacteriophage-originated polytubes as a carrier for the presentation of foreign sequences. Virus Res..

[bib0053] Špakova A., Šimoliūnas E., Batiuškaitė R., Pajeda S., Meškys R., Petraitytė-Burneikienė R. (2019). Self-assembly of tail tube protein of bacteriophage vB_EcoS_NBD2 into extremely long polytubes in E. Coli and S. cerevisiae. Viruses.

[bib72] Spohn G., Jennings G.T., Martina B.E., Keller I., Beck M., Pumpens P., Osterhaus A.D., Bachmann M.F. (2010). A VLP-based vaccine targeting domain III of the West Nile virus e protein protects from lethal infection in mice. Virol. J..

[bib0054] Tariq H., Batool S., Asif S., Ali M., Abbasi B.H. (2022). Virus-like particles: revolutionary platforms for developing vaccines against emerging infectious diseases. Front. Microbiol..

[bib0055] Truchado D.A., Rincón S., Zurita L., Ponz F., Kole C., Chaurasia A., Hefferon K.L., Panigrahi J. (2023). Tools & Techniques of Plant Molecular Farming.

[bib0056] Uhde-Holzem K., Fischer R., Commandeur U. (2007). Genetic stability of recombinant potato virus X virus vectors presenting foreign epitopes. Arch. Virol..

[bib0057] Urakami A., Sakurai A., Ishikawa M., Yap M.L., Flores-Garcia Y., Haseda Y., Aoshi T., Zavala F.P., Rossmann M.G., Kuno S., Ueno R., Akahata W. (2017). Development of a novel virus-like particle vaccine platform that mimics the immature form of alphavirus. Clin. Vaccine Immunol..

[bib0058] Varadi M., Anyango S., Deshpande M., Nair S., Natassia C., Yordanova G., Yuan D., Stroe O., Wood G., Laydon A., Zídek A., Green T., Tunyasuvunakool K., Petersen S., Jumper J., Clancy E., Green R., Vora A., Lutfi M., Figurnov M., Cowie A., Hobbs N., Kohli P., Kleywegt G., Birney E., Hassabis D., Velankar S. (2021). AlphaFold protein structure database: massively expanding the structural coverage of protein-sequence space with high-accuracy models. Nucleic Acids Res..

[bib0059] Wang Q., Kaltgrad E., Lin T., Johnson J.E., Finn M.G. (2002). Natural supramolecular building blocks: wild-type cowpea mosaic virus. Chem. Biol..

[bib0060] Ward B.J., Gobeil P., Séguin A., Atkins J., Boulay I., Charbonneau P.Y., Couture M., D'Aoust M.A., Dhaliwall J., Finkle C., Hager K., Mahmood A., Makarkov A., Cheng M.P., Pillet S., Schimke P., St-Martin S., Trépanier S., Landry N. (2021). Phase 1 randomized trial of a plant-derived virus-like particle vaccine for COVID-19. Nat. Med..

[bib0061] Wiedermann G., Scheiermann N., Goubau P., Ambrosch F., Gesemann M., De Bel C., Kremsner P., Paar D., Kunz C., Hauser P., Simoen E., Safary A., Andre F.E., Desmyter J. (1987). Multicentre dose range study of a yeast-derived hepatitis B vaccine. Vaccine.

[bib0062] Willyard C. (2017). The drug-resistant bacteria that pose the greatest health threats. Nature.

[bib0063] World Health Organization (2017).

[bib0064] Wu T., Li S.W., Zhang J., Ng M.H., Xia N.S., Zhao Q. (2012). Hepatitis E vaccine development: a 14-year odyssey. Hum. Vaccines Immunother..

[bib0065] Zamora-Ceballos M., Moreno N., Gil-Cantero D., Castón J.R., Blanco E., Bárcena J. (2022). Immunogenicity of multi-target chimeric RHDV virus-like particles delivering foreign B-cell epitopes. Vaccines.

[bib0067] Zimmermann L., Stephens A., Nam S.Z., Rau D., Kubler J., Lozajic M., Gabler F., Söding J., Lupas A.N., Alva V. (2018). A completely reimplemented MPI bioinformatics toolkit with a new HHpred server at its core. J. Mol. Biol..

[bib0068] Zinkhan S., Ogrina A., Balke I., Reseviča G., Zeltins A., de Brot S., Lipp C., Chang X., Zha L., Vogel M., Bachmann M.F., Mohsen M.O. (2021). The impact of size on particle drainage dynamics and antibody response. J. Control. Release.

[bib0069] Zvirbliene A., Samonskyte L., Gedvilaite A., Voronkova T., Ulrich R., Sasnauskas K. (2006). Generation of monoclonal antibodies of desired specificity using chimeric polyomavirus-derived virus-like particles. J. Immunol. Methods.

