# Supplementary material for: Immunogenicity of novel vB_EcoS_NBD2 bacteriophage-originated nanotubes as a carrier for peptide-based vaccines
Source: Virus Res. 2024 Apr 24;345:199370. doi: 10.1016/j.virusres.2024.199370 (PMC11059446; doi:10.1016/j.virusres.2024.199370)
Supplement: Supplementary file 1 — Supplementary Materials: Supplementary Table S1: Oligonucleotides used in this work; Supplementary Fig. S1: Expression vectors used for the synthesis of chimeric proteins in S. cerevisiae; Supplementary Fig. S2: Analysis of purified recombinant protein gp39 (a) by SDS-PAGE and (b) electron microscopy; Supplementary Fig. S3: Characterization of recombinant protein gp39 variants; Supplementary Table S2: The p-values of conducted immunological experiments in mice. [file mmc1.docx]

**Supplementary Table S1.** Oligonucleotides used in this work.

| **Primer name** | **Sequence (5' to 3')** | **Inserted restriction endonuclease site** |
| --- | --- | --- |
| Blp163_F | AGCCCGGGGATTCGTTTTTGGAAAGTGGTC | SmaI |
| Blp91_F | AGCCCGGGAATGGACATGACACGGTGTTAG | SmaI |
| Blp55_F | AGCCCGGGAGCGCATTAGCTAAATTTATTACTG | SmaI |
| Blp_R | GTGGATCCAACAATAATTTGCTGGTTGTTTAG | BamHI |
| Omp28_F | AGCCCGGGGGTTACACTTTCCAAGACACTC | SmaI |
| Omp28_R | GTGGATCCTAAATCGTCTTGTAATTCAGGTCCG | BamHI |
| Omp14_F | GGGAAAGGTGATGTAGACGGTCTTGCAGCTGGCGCTGAATACAAGG | SmaI |
| Omp14_R | GATCCCTTGTATTCAGCGCCAGCTGCAAGACCGTCTACATCACCTTTCCC | BamHI |

Restriction endonuclease recognition sites are underlined.


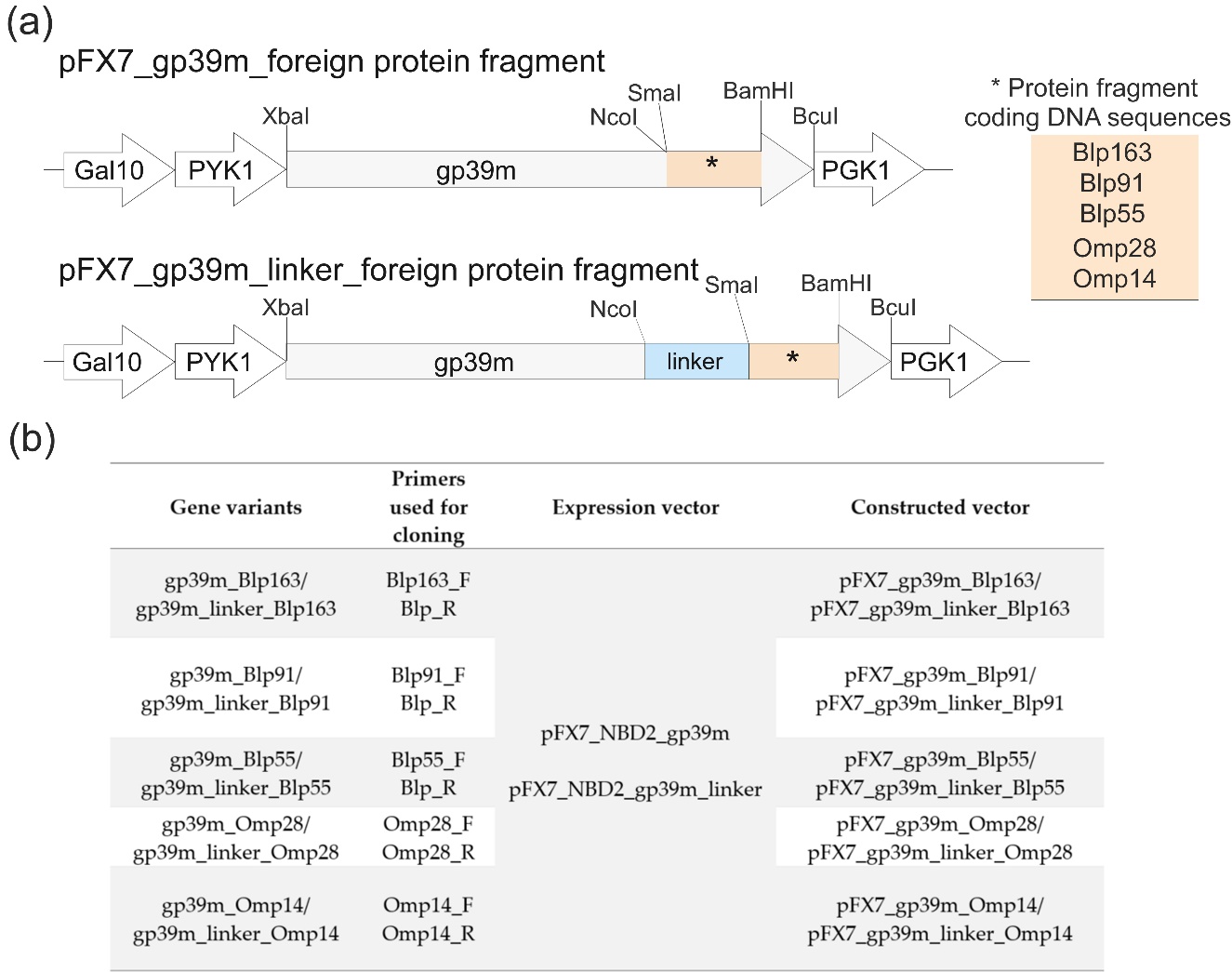


**Supplementary Fig. S1.** **Expression vectors used for the synthesis of chimeric proteins in
*S. cerevisiae.*** **(a)** A schematic representation of expression vectors used for the insertion of protein fragment-encoding DNA sequences. “Gal10-PYK1” – hybrid galactose-inducible promoter, “PGK1” – transcription terminator. In light blue color – linker sequence encoding glycine-serine aa repeat (GGGGS)_3x_, in light pink color – Blp1 and OmpA protein fragment encoding DNA sequences, “gp39m” represents modified gene sequence with a cloning site of NcoI, SmaI, BamHI and BcuI recognition sites. The image from Špakova et al. (2020) was reused and modified with the permission of authors. **(b)** The list of the constructed vectors. DNA fragments encoding Blp1 and OmpA protein fragments were named based on the length of the respective aa sequences.


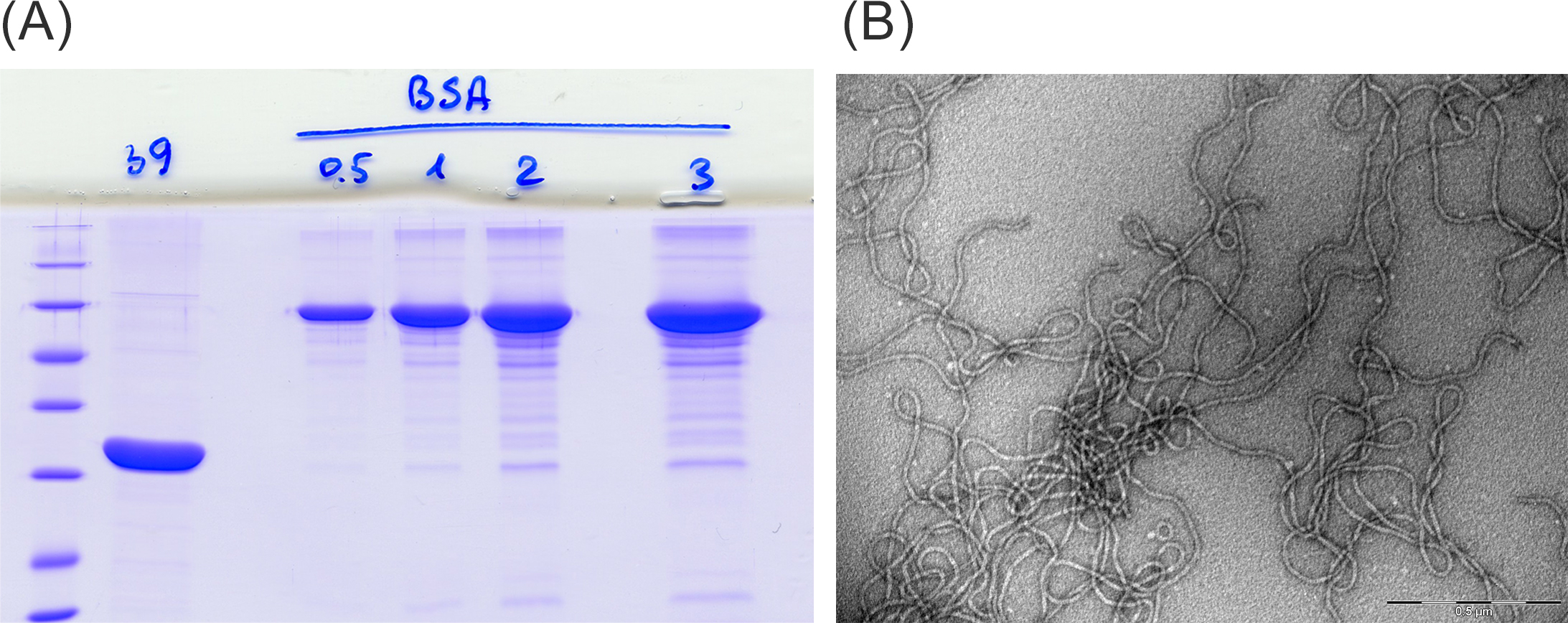


**Supplementary Figure S2. Analysis of purified recombinant protein gp39 (A) by SDS-PAGE and (B) electron microscopy.** **(A)** Purification efficiency of *S. cerevisiae-*derived recombinant protein gp39. First lane indicates Page ruler unstained protein ladder (Thermo Fisher Scientific, Vilnius, Lithuania); „39“ refers to purified recombinant protein gp39; “BSA” – bovine serum albumin, where the numbers refer their concentration in milligrams (mg). **(B)** Electron micrograph of gp39-derived polytubes. Scale bar represents 500 nm.


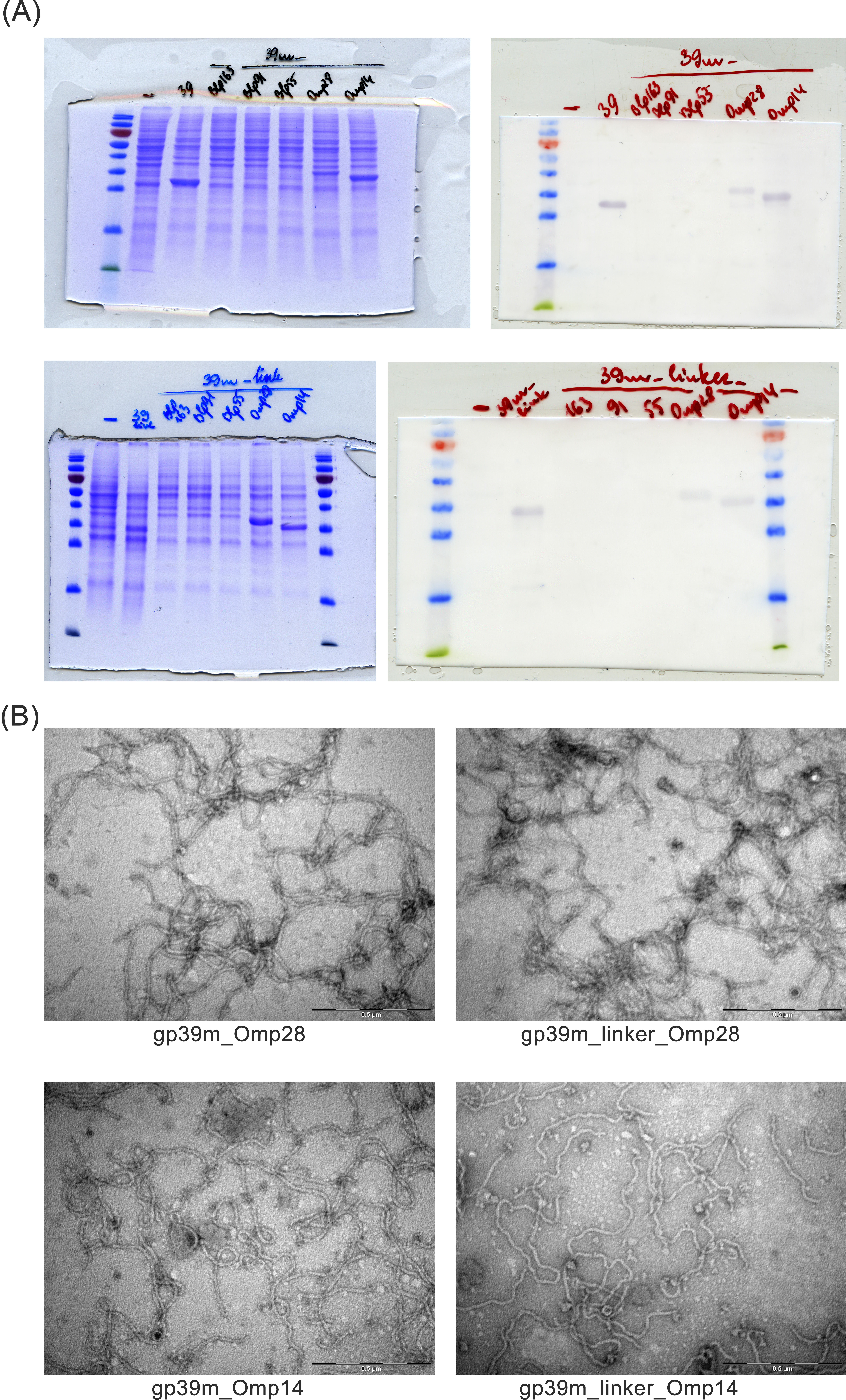


**Supplementary Figure S3.** **Characterization of recombinant protein gp39 variants.** **(A)** SDS-PAGE and Western blot analysis of yeast cell lysates. First lanes - page ruler prestained protein ladder (Thermo Fisher Scientifc, Vilnius, Lithuania). In the following lanes are fractionated cell lysates of a negative control (empty pFX7 vector-transformed yeast cells); recombinant protein gp39 or gp39m_linker and lysates with chimeric proteins having inserted foreign protein fragments of Blp163, Blp91, Blp55, Omp28, Omp14 into the C-terminus of the scaffold protein gp39m via a direct fusion or a ﬂexible glycine-serine linker (GGGGS)_3_. For Western blot analysis, gp39 protein-specific antibodies were used. **(B)** Electron micrographs of gp39-derived polytubes with inserted Omp28, Omp14 protein fragments. The scale bars indicate 500 nm.

**Supplementary Table S2.** The *p*-values of conducted immunological experiments in mice.

**(A)**

| **Comparison** | | | **p value** | **Significance** | **Used test** |
| --- | --- | --- | --- | --- | --- |
| Within mice group | 1st and 2nd: | gp39 | **0.04676** | **< 0.05** | *Wilcoxon Signed Rank* |
|  | 2nd and 3rd: | gp39 | 1.00 | > 0.05 |  |
|  | 1st and 2nd: | gp39 with adj. | 0.3458 | > 0.05 |  |
|  | 2nd and 3rd: | gp39 with adj. | 1.00 | > 0.05 |  |
| Two different mice groups | 1st: | gp39 *vs* gp39 with adj. | **0.009237** | **< 0.05** | *Mann-Whitney U* |
|  | 2nd: | gp39 *vs* gp39 with adj. | 0.1461 | > 0.05 |  |
|  | 3rd: | gp39 *vs* gp39 with adj. | 0.07528 | > 0.05 |  |
| Total evaluation between mice groups | gp39 *vs* gp39 with adj. | | **0.001012** | **< 0.05** |  |

**(B)**

| **Comparison** | | | | **p value** | **Significance** | **Used test** |
| --- | --- | --- | --- | --- | --- | --- |
| Within mice group | Antibodies against gp39 | 1st and 2nd: | gp39 | **0.043** | **< 0.05** | *Wilcoxon Signed Rank* |
|  |  | 2nd and 3rd: | gp39 | 0.176 | > 0.05 |  |
|  |  | 1st and 2nd: | gp39m_link_Omp28 | **0.042** | **< 0.05** |  |
|  |  | 2nd and 3rd: | gp39m_link_Omp28 | 0.068 | > 0.05 |  |
|  |  | 1st and 2nd: | gp39 + Omp28 | **0.041** | **< 0.05** |  |
|  |  | 2nd and 3rd: | gp39 + Omp28 | 0.109 | > 0.05 |  |
|  | Antibodies against gp39m_link_ Omp28 | 1st and 2nd: | gp39 | **0,042** | **< 0.05** |  |
|  |  | 2nd and 3rd: | gp39 | 0.414 | > 0.05 |  |
|  |  | 1st and 2nd: | gp39m_link_Omp28 | **0.042** | **< 0.05** |  |
|  |  | 2nd and 3rd: | gp39m_link_Omp28 | 0.180 | > 0.05 |  |
|  |  | 1st and 2nd: | gp39 + Omp28 | **0.041** | **< 0.05** |  |
|  |  | 2nd and 3rd: | gp39 + Omp28 | 0.854 | > 0.05 |  |
|  | Antibodies against Omp28 | 1st and 2nd: | gp39 | - | - |  |
|  |  | 2nd and 3rd: | gp39 | - | - |  |
|  |  | 1st and 2nd: | gp39m_link_Omp28 | 0.461 | > 0.05 |  |
|  |  | 2nd and 3rd: | gp39m_link_Omp28 | 0.892 | > 0.05 |  |
|  |  | 1st and 2nd: | gp39 + Omp28 | - | - |  |
|  |  | 2nd and 3rd: | gp39 + Omp28 | **0.043** | **< 0.05** |  |
|  |  | 1st and 2nd: | Omp28 | - | - |  |
|  |  | 2nd and 3rd: | Omp28 | - | - |  |
| Total evaluation between mice groups | Antibodies against gp39 | gp39 *vs* gp39m_link_Omp28 | | 0.385 | > 0.05 | *Mann-Whitney U* |
|  |  | gp39 *vs* gp39 + Omp28 | | 0.736 | > 0.05 |  |
|  |  | gp39m_link_Omp28 *vs* gp39 + Omp28 | | 0.768 | > 0.05 |  |
|  | Antibodies against gp39m_link_ Omp28 | gp39 *vs* gp39m_link_Omp28 | | 0.220 | > 0.05 |  |
|  |  | gp39 *vs* gp39 + Omp28 | | 0.766 | > 0.05 |  |
|  |  | gp39m_link_Omp28 *vs* gp39 + Omp28 | | 0.395 | > 0.05 |  |
| Two different mice groups | Antibodies against Omp28 | 2nd: | gp39m_link_Omp28 *vs* gp39 + Omp28 | **0.037** | **< 0.05** |  |
|  |  | 3rd: | gp39 *vs*gp39m_link_Omp28 | **0.005** | **< 0.05** |  |
|  |  | 3rd: | gp39 *vs* gp39 + Omp28 | **0.005** | **< 0.05** |  |
|  |  | 3rd: | gp39 *vs*Omp28 | 0.519 | > 0.05 |  |
|  |  | 3rd: | gp39m_link_Omp28 *vs* gp39 + Omp28 | 0.073 | > 0.05 |  |
|  |  | 3rd: | gp39m_link_Omp28 vs Omp28 | 0.159 | > 0.05 |  |
|  |  | 3rd: | gp39 + Omp28 *vs* Omp28 | **0.008** | **< 0.05** |  |

**(A)** The *p*-values of gp39 protein-specific antibody titers in mice groups with or without the use of adjuvant. **(B)** The *p-*values of antibody titers against protein gp39, chimeric gp39m_link_Omp28 or peptide Omp28 within individual mice groups and between different mice groups. The value of p < 0.05 was considered statistically significant. Statistical analysis was calculated using SPSS Statistics 23.0 (SPSS, Armonk, NY, USA) using Wilcoxon Signed Rank and Mann-Whitney U tests.
